# Supplementary material for: Short-term coastal forest responses to a hurricane-scale freshwater and saltwater flooding experiment
Source: PLoS One. 2026 May 13;21(5):e0323584. doi: 10.1371/journal.pone.0323584 (PMC13170863; doi:10.1371/journal.pone.0323584)
Supplement: S1 Table — (DOCX) [file pone.0323584.s001.docx]

Table S1. Water application for the TEMPEST experiment.

| Event | Date | Saltwater pump on  (EDT) | Saltwater pump off  (EDT) | Saltwater total vol (L) | Freshwater pump on  (EDT) | Freshwater pump off  (EDT) | Freshwater total vol (L) |
| --- | --- | --- | --- | --- | --- | --- | --- |
| TEMPEST I | 22 Jun 2022 | 06:05 | 15:38 | 266,832 | 06:07 | 16:00 | 262,992 |
