# Supplementary figures and images for: Short-term coastal forest responses to a hurricane-scale freshwater and saltwater flooding experiment
Source: PLoS One. 2026 May 13;21(5):e0323584. doi: 10.1371/journal.pone.0323584 (PMC13170863; doi:10.1371/journal.pone.0323584)

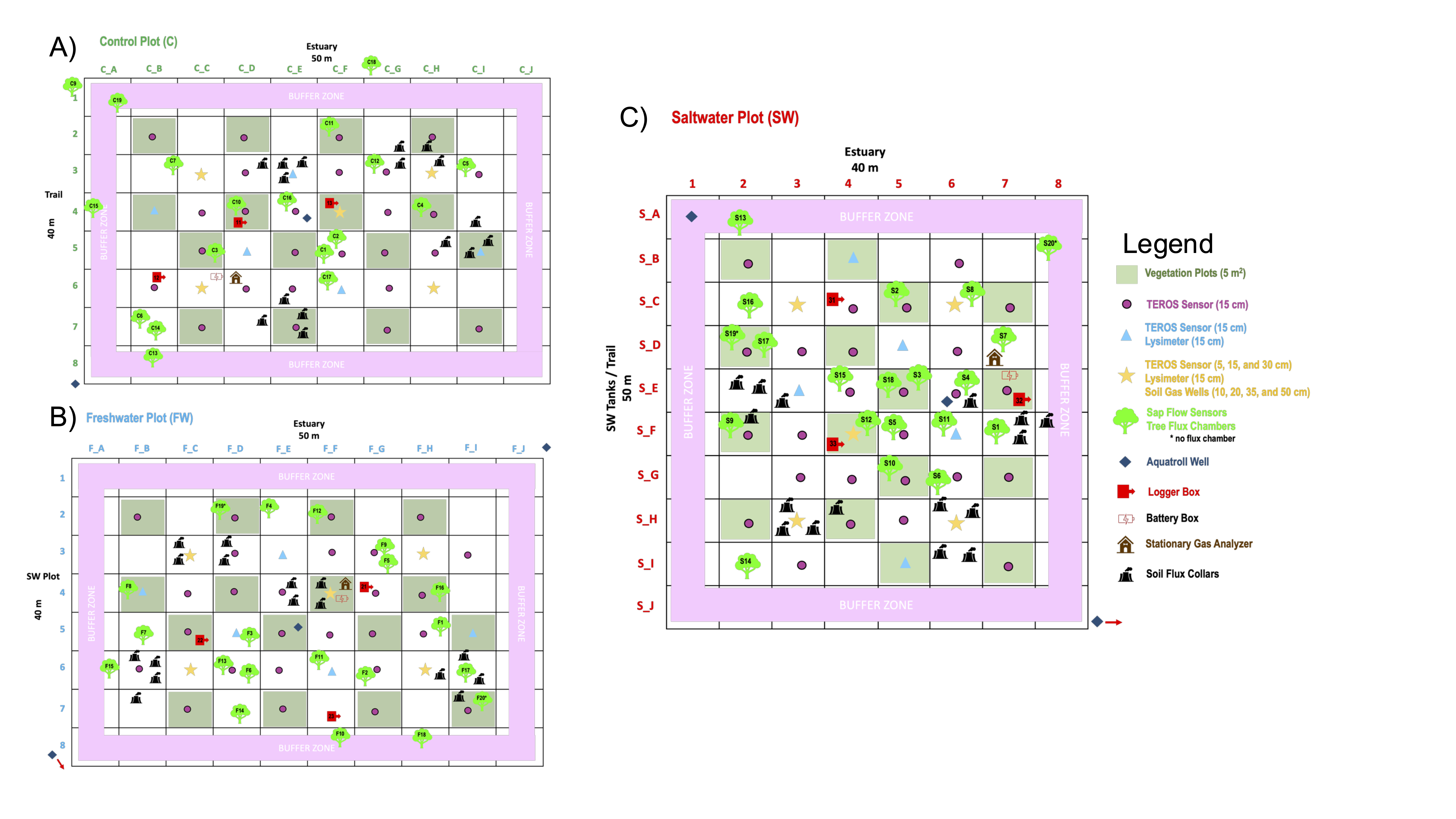

Supplement: S1 Fig — (PNG) [file pone.0323584.s002.png]

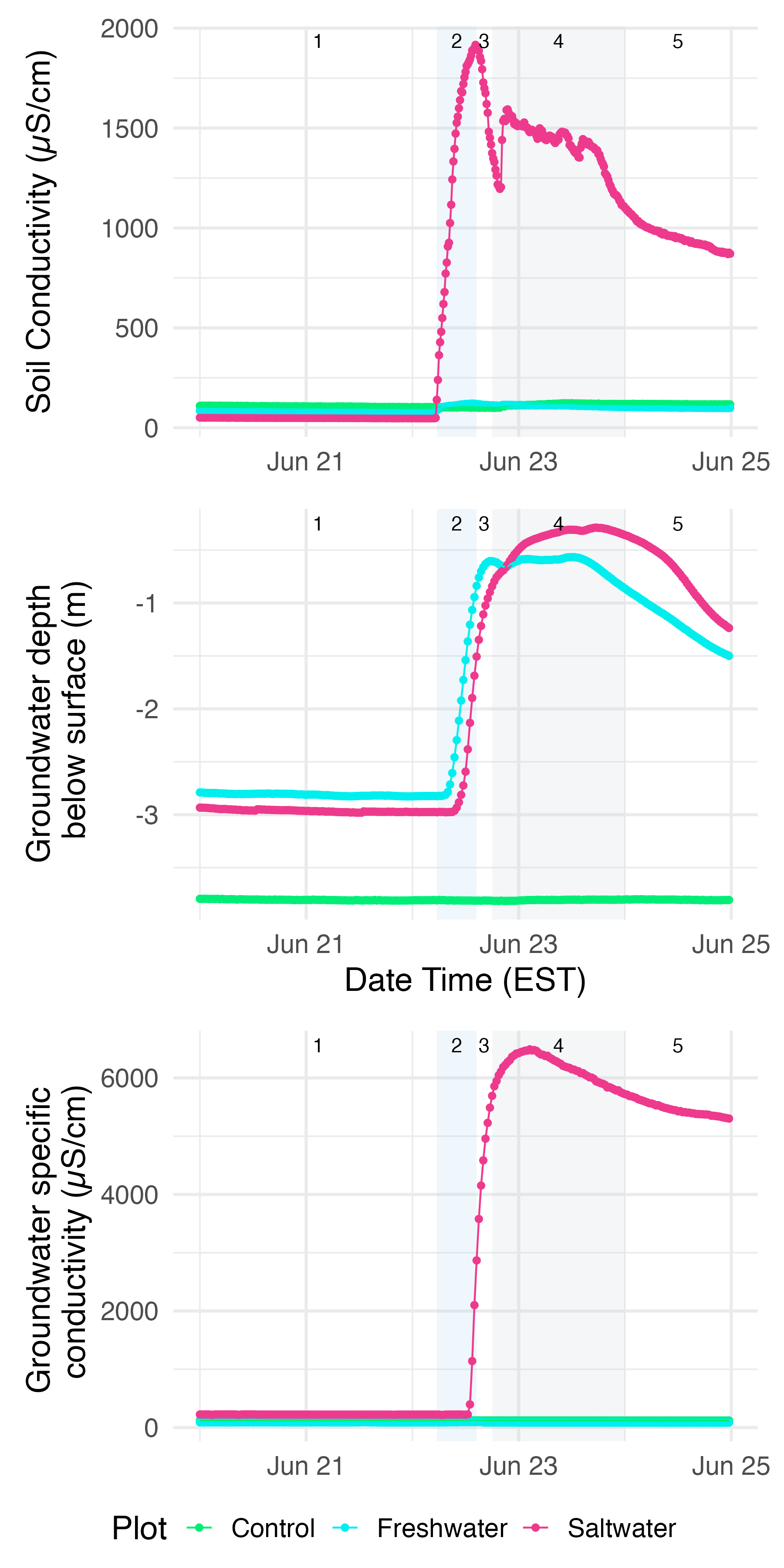

Supplement: S2 Fig — (PNG) [file pone.0323584.s003.png]

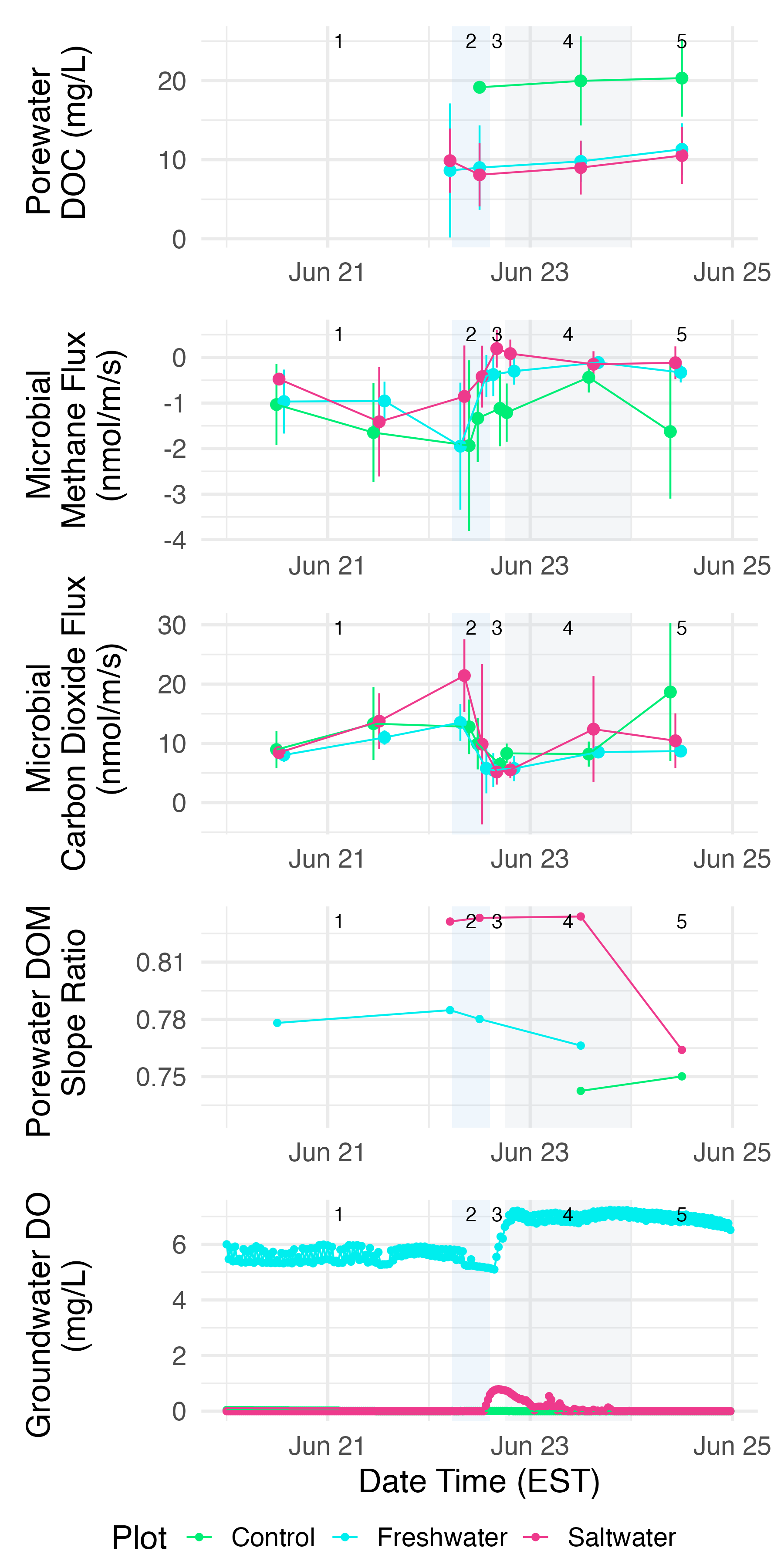

Supplement: S3 Fig — (PNG) [file pone.0323584.s004.png]

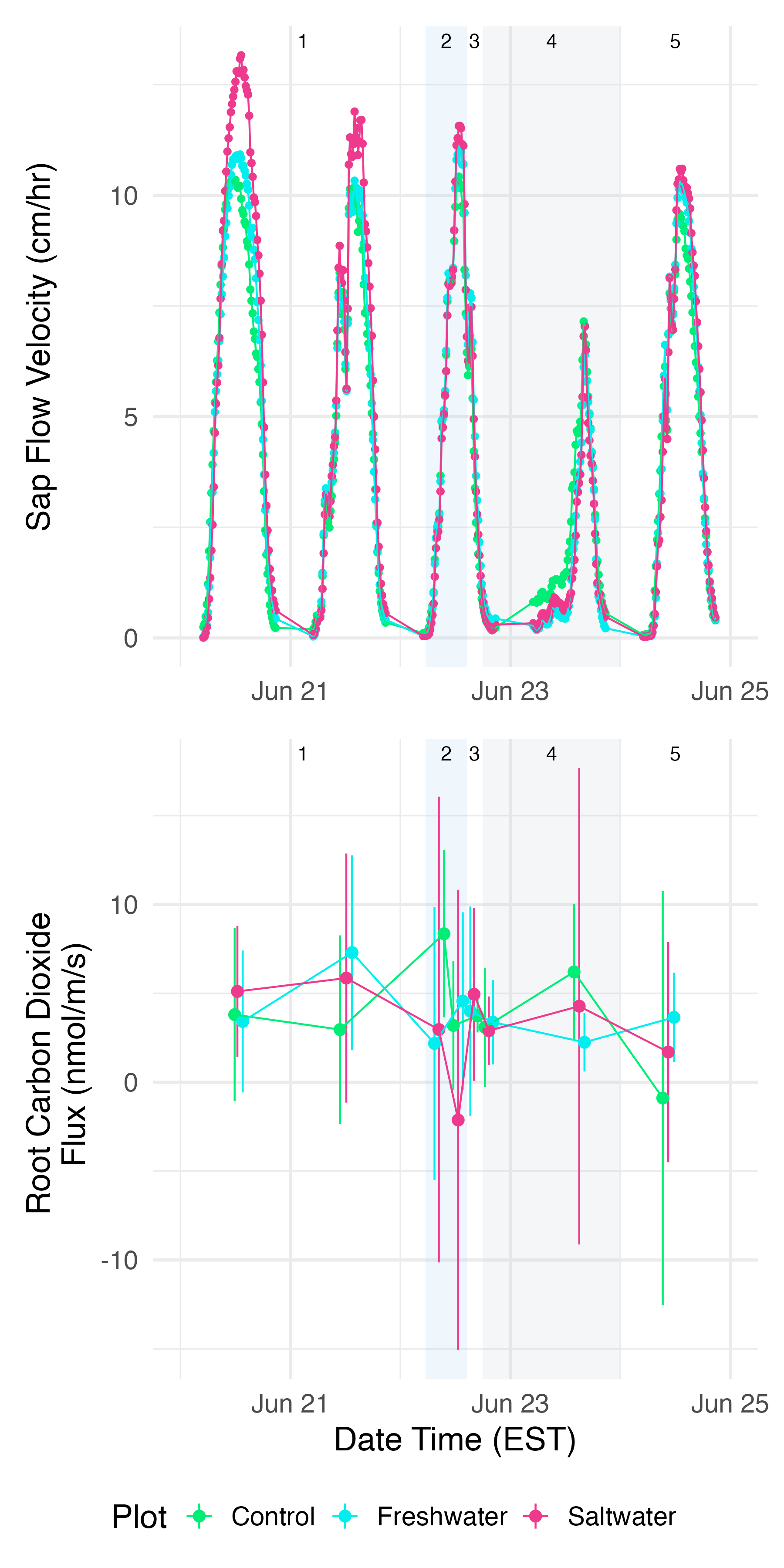

Supplement: S4 Fig — (PNG) [file pone.0323584.s005.png]
