# Supplementary material for: Short-term coastal forest responses to a hurricane-scale freshwater and saltwater flooding experiment
Source: PLoS One. 2026 May 13;21(5):e0323584. doi: 10.1371/journal.pone.0323584 (PMC13170863; doi:10.1371/journal.pone.0323584)
Supplement: Attachment — Submitted filename: Reviewer Responses.pdf [file pone.0323584.s006.pdf]

PONE-D-25-19393
Short-term coastal forest responses to a hurricane-scale freshwater and saltwater flooding experiment
PLOS ONE

Dear Dr. Myers-Pigg,

Thank you for submitting your manuscript to PLOS ONE. After careful consideration, we feel that it has merit but does not fully meet PLOS ONE’s publication criteria as it currently stands. Therefore, we invite you to submit a revised version of the manuscript that addresses the points raised during the review process.

==============================
ACADEMIC EDITOR:
Dear authors, thank you considering PLOS One. The study is interesting and within the scope of the Journal. However, based on the reviewers comments, the manuscript needs substantial changes. Please refer to the comments of each reviewer and address all queries.
==============================

> **Thank you for the opportunity to revise the manuscript. We provide responses to each reviewer/editor comment in bold, black, indented text below.**

Please submit your revised manuscript by Nov 22 2025 11:59PM. If you will need more time than this to complete your revisions, please reply to this message or contact the journal office at plosone@plos.org. When you're ready to submit your revision, log on to https://www.editorialmanager.com/pone/ and select the 'Submissions Needing Revision' folder to locate your manuscript file.

Please include the following items when submitting your revised manuscript:

- A rebuttal letter that responds to each point raised by the academic editor and reviewer(s). You should upload this letter as a separate file labeled 'Response to Reviewers'.
- A marked-up copy of your manuscript that highlights changes made to the original version. You should upload this as a separate file labeled 'Revised Manuscript with Track Changes'.
- An unmarked version of your revised paper without tracked changes. You should upload this as a separate file labeled 'Manuscript'.

If you would like to make changes to your financial disclosure, please include your updated statement in your cover letter. Guidelines for resubmitting your figure files are available below the reviewer comments at the end of this letter.

If applicable, we recommend that you deposit your laboratory protocols in protocols.io to enhance the reproducibility of your results. Protocols.io assigns your protocol its own identifier (DOI) so that it can be cited independently in the future. For instructions see: https://journals.plos.org/plosone/s/submission-guidelines#loc-laboratory-protocols. Additionally, PLOS ONE offers an option for publishing peer-reviewed Lab Protocol articles, which describe protocols hosted on protocols.io. Read more information on sharing protocols at https://plos.org/protocols?utm_medium=editorial-email&utm_source=authorletters&utm_campaign=protocols.

We look forward to receiving your revised manuscript.

Kind regards,

Miguel Inácio
Academic Editor
PLOS ONE

Journal requirements:

When submitting your revision, we need you to address these additional requirements.

1. Please ensure that your manuscript meets PLOS ONE's style requirements, including those for file naming. The PLOS ONE style templates can be found at

https://journals.plos.org/plosone/s/file?id=wjVg/PLOSOne_formatting_sample_main_body.pdf and

https://journals.plos.org/plosone/s/file?id=ba62/PLOSOne_formatting_sample_title_authors_affiliations.pdf

2. In your Methods section, please provide additional information regarding the permits you obtained for the work. Please ensure you have included the full name of the authority that approved the field site access and, if no permits were required, a brief statement explaining why.

The following statement has been added to the Methods section lines 174-175:

“Site access was provided by the Smithsonian Institution and a permit to extract the seawater from the Rhode River Estuary was provided by the Maryland Department of the Environment.”

3. Thank you for stating the following financial disclosure:

“COMPASS-FME, a multi-institutional project supported by the U.S. Department of Energy, Office of Science, Biological and Environmental Research as part of the Environmental System Science Program.”

Please state what role the funders took in the study. If the funders had no role, please state: "The funders had no role in study design, data collection and analysis, decision to publish, or preparation of the manuscript."

If this statement is not correct you must amend it as needed.

Please include this amended Role of Funder statement in your cover letter; we will change the online submission form on your behalf.

This information has been added to our cover letter.

4. Thank you for stating the following in the Acknowledgments Section of your manuscript:

“We thank the massive field sampling and infrastructure teams that made this first TEMPEST event possible – in particular, Rick Smith, Stella Woodard and Evan Phillips. We also thank James Stegen for his contributions to early conceptualization of the TEMPEST experimental design. This research was supported by COMPASS-FME, a multi-institutional project supported by the U.S. Department of Energy, Office of Science, Biological and Environmental Research as part of the Environmental System Science Program, and by the Smithsonian Environmental Research Center. The Pacific Northwest National Laboratory is operated for DOE by Battelle Memorial Institute under contract DE-AC05-76RL01830.”

We note that you have provided funding information that is currently declared in your Funding Statement. However, funding information should not appear in the Acknowledgments section or other areas of your manuscript. We will only publish funding information present in the Funding Statement section of the online submission form.

Please remove any funding-related text from the manuscript and let us know how you would like to update your Funding Statement. Currently, your Funding Statement reads as follows:

“COMPASS-FME, a multi-institutional project supported by the U.S. Department of Energy, Office of Science, Biological and Environmental Research as part of the Environmental System Science Program.”

Please state what role the funders took in the study. If the funders had no role, please state: "The funders had no role in study design, data collection and analysis, decision to publish, or preparation of the manuscript."

Please include your amended statements within your cover letter; we will change the online submission form on your behalf.

> **This information has been removed from the manuscript text and added to our cover letter.**

5. When completing the data availability statement of the submission form, you indicated that you will make your data available on acceptance. We strongly recommend all authors decide on a data sharing plan before acceptance, as the process can be lengthy and hold up publication timelines. Please note that, though access restrictions are acceptable now, your entire data will need to be made freely accessible if your manuscript is accepted for publication. This policy applies to all data except where public deposition would breach compliance with the protocol approved by your research ethics board. If you are unable to adhere to our open data policy, please kindly revise your statement to explain your reasoning and we will seek the editor's input on an exemption. Please be assured that, once you have provided your new statement, the assessment of your exemption will not hold up the peer review process.

> **Data and codes are available at:**
> **Myers-Pigg A; Hopple A; Pennington S C; Regier P; Bond-Lamberty B; DiCianna M J; Doro K O; McDowell N; McElhinny J; Stearns A; Ward N; Bailey V; Megonigal J P (2025): Data for Myers-Pigg et al. (submitted), "Short-term coastal forest responses to a hurricane-scale freshwater and saltwater flooding experiment". COMPASS-FME. Dataset. doi:10.15485/2999515**

6. One of the noted authors is a group or consortium [TEMPEST 1.0 Event Consortium]. In addition to naming the author group, please list the individual authors and affiliations within this group in the acknowledgments section of your manuscript. Please also indicate clearly a lead author for this group along with a contact email address.

The consortium list has been modified to include affiliations for the authors listed.

7. We note that Figure 1 in your submission contain copyrighted images. All PLOS content is published under the Creative Commons Attribution License (CC BY 4.0), which means that the manuscript, images, and Supporting Information files will be freely available online, and any third party is permitted to access, download, copy, distribute, and use these materials in any way, even commercially, with proper attribution. For more information, see our copyright guidelines: http://journals.plos.org/plosone/s/licenses-and-copyright.

We require you to either (1) present written permission from the copyright holder to publish these figures specifically under the CC BY 4.0 license, or (2) remove the figures from your submission:

1. You may seek permission from the original copyright holder of Figure 1 to publish the content specifically under the CC BY 4.0 license.

We recommend that you contact the original copyright holder with the Content Permission Form (http://journals.plos.org/plosone/s/file?id=7c09/content-permission-form.pdf) and the following text:

“I request permission for the open-access journal PLOS ONE to publish XXX under the Creative Commons Attribution License (CCAL) CC BY 4.0 (http://creativecommons.org/licenses/by/4.0/). Please be aware that this license allows unrestricted use and distribution, even commercially, by third parties. Please reply and provide explicit written permission to publish XXX under a CC BY license and complete the attached form.”

Please upload the completed Content Permission Form or other proof of granted permissions as an "Other" file with your submission.

In the figure caption of the copyrighted figure, please include the following text: “Reprinted from [ref] under a CC BY license, with permission from [name of publisher], original copyright [original copyright year].”

2. If you are unable to obtain permission from the original copyright holder to publish these figures under the CC BY 4.0 license or if the copyright holder’s requirements are incompatible with the CC BY 4.0 license, please either i) remove the figure or ii) supply a replacement figure that complies with the CC BY 4.0 license. Please check copyright information on all replacement figures and

update the figure caption with source information. If applicable, please specify in the figure caption text when a figure is similar but not identical to the original image and is therefore for illustrative purposes only.

We apologize for the confusion about figure 1, which is an original creation for this submission, although panel 1 was inspired by a photograph in Hopple et al. We've modified the language to state that the figure is similar but not identical to the original image (Lines 135-145).

If the reviewer comments include a recommendation to cite specific previously published works, please review and evaluate these publications to determine whether they are relevant and should be cited. There is no requirement to cite these works unless the editor has indicated otherwise.

[Note: HTML markup is below. Please do not edit.]

Reviewers' comments:

Reviewer's Responses to Questions

Comments to the Author

1. Is the manuscript technically sound, and do the data support the conclusions?

The manuscript must describe a technically sound piece of scientific research with data that supports the conclusions. Experiments must have been conducted rigorously, with appropriate controls, replication, and sample sizes. The conclusions must be drawn appropriately based on the data presented.

Reviewer #1: Yes

Reviewer #2: No

Reviewer #3: Partly

---

2. Has the statistical analysis been performed appropriately and rigorously?

Reviewer #1: I Don't Know

Reviewer #2: No

Reviewer #3: Yes

---

3. Have the authors made all data underlying the findings in their manuscript fully available?

The PLOS Data policy requires authors to make all data underlying the findings described in their manuscript fully available without restriction, with rare exception (please refer to the Data Availability Statement in the manuscript PDF file). The data should be provided as part of the manuscript or its supporting information, or deposited to a public repository. For example, in addition to summary statistics, the data points behind means, medians and variance measures should be available. If there are restrictions on publicly sharing data—e.g. participant privacy or use of data from a third party—those must be specified.

Reviewer #1: Yes

Reviewer #2: Yes

Reviewer #3: Yes

---

4. Is the manuscript presented in an intelligible fashion and written in standard English?

PLOS ONE does not copyedit accepted manuscripts, so the language in submitted articles must be clear, correct, and unambiguous. Any typographical or grammatical errors should be corrected at revision, so please note any specific errors here.

Reviewer #1: Yes

Reviewer #2: Yes

Reviewer #3: No

---

5. Review Comments to the Author

Please use the space provided to explain your answers to the questions above. You may also include additional comments for the author, including concerns about dual

publication, research ethics, or publication ethics. (Please upload your review as an attachment if it exceeds 20,000 characters)

Reviewer #1: Summary
This manuscript reports data collected during early stages of the TEMPEST project which aims to better understand mechanisms driving upland forest conversion through fresh and saltwater flooding events that are increasing in frequency. They measure a suite of hydrologic, biogeochemical and vegetation variables that can indicate change in ecosystem function before trees are visibly dying and therefore may provide a greater understanding of the mechanisms that result in ghost forest formation and marsh encroachment. Although authors provide motivation for the larger study, it is not clear what can be learned from looking at the data presented here which was collected after the first of many planned flooding events. More specifics could be included in the introduction to motivate this study in isolation from the larger TEMPEST project to make it a more compelling manuscript. Below I provide general comments followed by more specific line-by-line comments about this overarching concern.

> **We thank the reviewer for pointing out the opportunity for us to strengthen the narrative throughout the manuscript. In addition to addressing the specific comments from the review below, we have made several overarching substantive changes throughout the manuscript focused on increasing motivation for the data presented in this manuscript to address this comment:**
>
> 1) **Added a paragraph in the introduction discussing novel disturbances and disturbance legacies (Lines 91-108)**
> 2) **Clarification on study hypotheses and their rationale (Lines 111-120)**
> 3) **Modified the paragraph on study motivation to include more rationale related to the first event (Lines 109-129).**
> 4) **Removal of discussion related to the overarching experiment in the discussion section.**

General comments

Abstract

• It would be helpful to include some indication of the flooding treatment that you are referring to and how it allows you to decouple the impacts of freshwater flooding and saltwater exposure on upland forests.

We have added on line 46 the word "experimental" before the first instance "flooding" to help clarify this point.

• The final sentence of the abstract reflects a larger issue with the manuscript. Beyond just suggesting that fore frequent/sustained flooding events are needed to drive more significant change, what can be learned the lack of change in many measured variables in response to this first flooding event?

Per our response to your high level summary, we've made substantial changes to the framing of the study to focus more on the first event and less on the overall experiment. We have also reworded the final abstract sentence to the two following sentences, which focus on the first flooding event:

"However, we show that even a single episodic event can cause large transient shifts in belowground conditions that drive physiological changes in coastal forest functions, such as soil moisture and oxygen levels. Such responses may impact how the system responds to future perturbations."

Introduction

• The introduction could use some more specific information to provide context for your hypotheses and the suite of measurements included in this study. Some examples of missing information I think could improve the introduction:
o L59: What are the 'hydrologic extremes' you are referring to and what is driving increases in the frequency of their occurrence?

On line 62, we added examples of hydrologic extremes we are referring to. What is driving them is discussed later in the paragraph on lines 69-72.

o What are the broader implications of studying hydrologic shifts and upland forest conversion? Why is this study important (beyond the general statement about improving modelling)? Could be coming at this from the angle of carbon storage, greenhouse gas production, habitat availability, etc…

We acknowledge that there are multiple reasons that this study would be of interest/important, such as the reasons listed above by the reviewer. We chose to include a specific example of land management decision making in the manuscript text as motivation. This sentence now reads:

“ The mechanisms that lead to coastal ecosystem state change are important to understand for myriad reasons, including informing land management decisions [1], particularly as the frequency, intensity, and rate of such changes accelerate due to changing climate [19].”

o What specific shifts in belowground biogeochemistry are expected and how might those lead to shifts in vegetation? There is currently no context for the specific measurements mentioned in Table 1 and outlined in the methods section.

We have added in details to the paragraph linking above and belowground processes (lines 76-90). Please see the response to the next comment for detailed examples of the changes made.

o L72-80: Could be replaced with a more specific explanation of the gaps in knowledge that past studies have not yet filled. This paragraph is currently very vague.

This comment relates to the previous one, and we’ve made alterations to this paragraph and the following one to incorporate changes in response to both comments. This paragraph now includes ties to the measurements in Table 1 and links between above and belowground processes (Lines 83-86). We also include reference to the high level knowledge gap this study is designed to address on lines 101-103. Further, we include a specific rationale for the current study that highlights knowledge gaps, lines 107-108 .

o Clarify the importance of separating the effects of freshwater and saltwater flooding?

We have added discussion of the importance of separating out freshwater and saltwater flooding in lines 127-129.

o Hypothesis statements are also very broad. Would it be possible to have the hypothesis statements more directly address specific mechanisms related to the potential impacts of flooding a system for the first time (as opposed to chronic flooding that will be tested later in the experiment)? Are there specific metrics that are expected to change early in the experiment and therefore act as early signals of forest conversion?

We have increased the specificity of the timescales of our hypotheses. We also discuss why the hydrologic, biogeochemical, and vegetation variables were chosen, and highlight an example of how belowground biogeochemistry may act as early indicators of forest stress (lines 109-131).

Discussion

• Similar concern as was expressed for the abstract and introduction: most of the discussion is focused on transient or non-significant changes that could be more important with increased duration/frequency of flooding later in the experiment. Did we learn anything about forest conversion from looking at results from the first flooding event in isolation?

This study finds that a single flooding event is not enough to cause forest conversion. This is consistent with the results from our second year of results recently published (Reiger et al., 2025), but that study did not explore first-year responses in any depth. We have reduced the number of conjectures about longer term impacts throughout the discussion to emphasize the importance of studying this first event and the need to understand belowground biogeochemical changes before aboveground shifts are seen.

Regier P, Bond-Lamberty B, Ward N, Bailey V, Peixoto RB, Machado-Silva F, et al. Short-term experimental flooding impacts soil biogeochemistry but not aboveground vegetation in a coastal forest. Proc Natl Acad Sci U S A. 2025;122: e2511756122.

Line by line comments:

• L60: might be helpful to indicate that formation of ghost forests is driven by tree mortality

We have worked this into the existing sentence, which now reads:

"The upland boundaries of coastal ecosystems are becoming increasingly exposed to hydrological extremes such as storm surge, coastal flooding, extreme rain events, and hurricanes, causing ecophysiological stress that over time can dramatically transform upland forests through increased tree mortality to 'ghost forests' and then wetlands [1]."

• L98: This is the first time you mention pulse and press disturbance. I would suggest removing this terminology or describing the concept in more detail.

We now provide details on lines 91 and 101 what we mean by press and pulse.

• L81-82: I would suggest providing some indication of your experimental design here given that you have highlighted that your methods are novel and therefore an important part of understanding the importance of your work.

This sentence now reads:

"Here we consider the effects of novel flooding on a coastal upland forest by experimentally flooding large forest plots (2000 m^2) with freshwater and saline water to achieve transient soil saturation [27]."

• L74-76: What are you referring to when you say "this is particularly true"? I would suggest being more specific. What types of shifts in belowground biogeochemistry do you expect to observe?

We have edited this paragraph significantly to be more specific about the belowground biogeochemical shifts expected (lines 81-83).

• L82-85: Specify the timescale are you referring to in this hypothesis.

We've modified this sentence to read:

"While we expect that the effects of short-term applications will begin small and increase with successive exposures (Fig 1), we also hypothesize that hydrologic state variables (e.g., soil moisture and groundwater levels) will respond to the event immediately but transiently, followed by muted biogeochemical (e.g., soil greenhouse gases, soil porewaters) responses, and finally minimal vegetation (e.g., tree sap flux density) responses in the 48 hours following the event."

• L 89: What do you specifically mean by 'experimental flooding'? This might be a good place to very briefly introduce the flooding treatments used in your study.

We now introduce the concept at the start of the paragraph lines 109-111.

• Figure 1: Provide more explanation of the blue line and boxes in Figure 1b. What is the frequency of water pulse events and does it change through time? Clarify that data in this ms is coming from after the first flooding event (blue box 1?), and future work will

assess how effects of flooding change with subsequent flooding events. Is ‘Unseen ecological responses’ indicating a hypothesis or result?

We have edited the figure 1 caption. It now reads:

Fig 1. a) Site and experimental design, inspired by [28]. b) The goal of the long-term manipulations is to stimulate changes in ecosystem state. The blue lines and waves represent each planned flooding event, with the first event described herein denoted as “1”. Each planned event will increase the frequency of flooding, i.e., the second event will flood the plots for 10 hours on each of 2 consecutive days, the third floods the plots for 10 hours on each of 3 consecutive days, etc. Panel b is inspired by [36]. Zooming into the first event described here, we focus on vegetation, biogeochemical and hydrological variables, changes to which are not directly observable aboveground. Note that we employ an adaptive experimental design, where we make necessary changes to infrastructure and timing of events based on what we’ve learned in previous years. Therefore, the blue waves and lines do not depict the exact details of future events.

• L131: How frequent are the flooding events?

We have added the following sentence on lines 169-170:

“ Each flooding event is targeted to last 10 hours, and each year the number of events is increased (Fig 1). “

• L191-197: When were these gas measurements collected in relation to the flooding event. How frequently?

Thanks for highlighting that there were some key details missing from our methods sections; we’ve added high level details on timing of sampling to our methods section throughout. For gas measurements specifically, the following sentence has been added in line 237-238:

“Samples were collected the day before, during, and for several days after the event [39].”

• L 207-208: There is currently no context for understanding the potential for roots to influence CH4 fluxes.

We've added these details in the introduction about why the measurements were included on lines 122-127.

• L250-251: How are you assessing differences in flooding response between fresh and saltwater plots when this is an unreplicated experiment?

The paragraph following the one called out in this comment was meant to describe how we are assessing the differences in plots. However, we recognize that the way this was presented may have led to some confusion. We have clarified this section. In short, we are conducting ANOVAs on a metric that normalizes each response relative to their values before the event (i.e., the change variable); such an approach is standard in the literature (e.g. Patrick et al., 2020). We also provide details on our statistical tests in our R processing scripts (`tsla_stats.Rmd`; lines 52-61 & lines 139-145) **in our data package.**

Patrick CJ, Yeager L, Armitage AR, Carvallo F, Congdon VM, Dunton KH, et al. A system level analysis of coastal ecosystem responses to hurricane impacts. Estuaries Coasts. 2020;43: 943–959.

• L290-292: This statement is confusing – consider revising to clarify the takeaway from this finding.

We have modified this statement. It now reads:

"There were no differences in variables relative to their values before the event (i.e., change) between the treatment plots (freshwater and saltwater, $p = 0.557$). However, we did find that the changes in variables relative to their values before the event differed across system components ($p <0.05$)."

• Figure 4: Not intuitive to see x axes on the top rather than the bottom of the figures.

We understand that this display of data may not be intuitive to all readers. It is common in soil sciences, however, where soil profiles are commonly represented with 0 as the soil surface and variables varying with depth below the soil surface. The graph reflects the physical distribution of the variables which makes it intuitive to think about in comparison to soil profiles. Therefore, we've elected to keep the figure the same.

• Table 2: Seems like this could be better shown in a graph rather than table.

> **We appreciate this suggestion; however, some of this text refers to Figure 2, while the time series plots for these data are our entire supplemental materials! We believe this is too much to include in one figure. However, based on this reviewer suggestion, we now direct readers to the relevant figures within the table caption itself.**

• Lines 363-364, 368-369, etc (throughout results): Should changes that are smaller than variability measured in the control plots be reported as change? It seems more accurate to say that neither saltwater nor freshwater flooding drove significant change in porewater DOC/microbial CH4 flux….

> **The only statistical tests we did were the ANOVAs discussed in our response to** L250-251 **above. Therefore, we do not use "significant" terminology outside of the statistical tests performed in the manuscript. However, we have modified our language through the results section to make our results clearer. We focused on clarifying our language for when variables were or were not outside the control plot variance window during the event, and discussed notable trends when they existed during or after the event.**

• L374 (and Table 2): The first time 'lagged' terminology is introduced. It would help to have context for understanding why timing of responses to flooding may differ between variables and why this could be important?

> **Thank you for pointing this out. We now introduce the lagged concept and terminology in the introduction on lines 87. We also increase the description of temporal trends in responses throughout the results section.**

• L494-496: I see this concept underlying the importance of your current study (i.e. responses of an upland forest to a single flooding event) and could be introduced much earlier and more explicitly.

> **Thanks for the suggestion! We have created a new paragraph (lines 91-108) in the introduction on the importance of understanding disturbance legacies to introduce this topic.**

• L502-504: A shortcoming of the study that should be acknowledged as such.

We believe that this comment is in reference to the part of the original statement on O2 responses being due to differences in hydrology between the plots. We've expanded the discussion of plot level heterogeneity (lines 495-500).

• L509-524: Not sure this paragraph belongs in the discussion section because it doesn't include any discussion of results being presented in this manuscript and instead is just plugging the importance of the larger project.

We agree that this does not include any new discussion of the results, but we do think that the concepts are useful context for framing the importance of our study. However, we agree that the original topic sentence read like a plug for the larger project and have removed it. We moved the remainder of the paragraph up into the introduction, and include discussion of the future TEMPEST events in the new "Conclusions and Future Research Directions" section.

Reviewer #2: The authors present an experiment designed to test the effects of a pulse disturbance on hydrologic, biogeochemical, and vegetation components in a coastal upland forest. While the study addresses an important topic, it suffers from several significant weaknesses that prevent it from being suitable for publication in its current form.

One of my primary concerns is the lack of replication in the experimental design, which severely undermines the validity of the results and the reliability of the conclusions drawn. Without replication, it is difficult to distinguish treatment effects from background variability. Secondly, the scientific relevance of the experiment is questionable. As the authors rightly point out in the introduction, we are facing a context of rapidly changing climate. Given this, an experimental design that investigates high-frequency or chronic flooding events would be of greater relevance and value to the scientific community than a single pulse disturbance. I would strongly recommend rethinking the experimental setup to test different climate change scenarios, particularly in relation to the increasing frequency and intensity of storm surges.

For these reasons, I regret to recommend rejection of this manuscript for publication.

We appreciate the reviewer's perspective and acknowledge there is a strong propensity in ecological research for replicated experimental designs. However, unreplicated designs are also common, particularly in large-scale experiments that cannot be replicated due to logistical

constraints (e.g. Biosphere 2; See excellent discussion on this topic in Caldararu et al., 2023 on this topic). Ecologists and biogeochemists in particular often use this approach to study impacts of perturbation in both aquatic and terrestrial ecosystems (see discussion and references in Hopple et al., 2023). The rich history of important discoveries and insights from unreplicated designs includes watershed manipulation studies at Hubbard Brook that began in the 1950s and is still yielding important and novel discoveries (Campbell et al., 2021). These ecosystem-scale manipulations have also been previously discussed as a positive example of experimental design in the important article on pseudoreplication by Hurlbert, 1984.

Caldararu, S., Rolo, V., Stocker, B. D., Gimeno, T. E., and Nair, R.: Ideas and perspectives: Beyond model evaluation – combining experiments and models to advance terrestrial ecosystem science, Biogeosciences, 20, 3637–3649, https://doi.org/10.5194/bg-20-3637-2023, 2023.

Campbell, J.L., Rustad, L.E., Bailey, S.W., Bernhardt, E.S., Driscoll, C.T., Green, M.B., Groffman, P.M., Lovett, G.M., McDowell, W.H., McGuire, K.J. and Rosi, E.J., 2021. Watershed studies at the Hubbard Brook Experimental Forest: Building on a long legacy of research with new approaches and sources of data. *Hydrological Processes*, *35*(1), p.e14016.

Hopple AM, Doro KO, Bailey VL, Bond-Lamberty B, McDowell N, Morris KA, et al. Attaining freshwater and estuarine-water soil saturation in an ecosystem-scale coastal flooding experiment. Environ Monit Assess. 2023;195: 425.

Hurlbert, S.H. 1984. Pseudoreplication and the design of ecological field experiments. *Ecological monographs, 54(2)*, pp. 187-211.

Reviewer #3: Manuscript Title: Short-term coastal forest responses to a hurricane-scale freshwater and saltwater flooding experiment
In this research, the authors studied freshwater and saltwater flooding in the coastal region. The study is important but needs extensive revision before publication.

1. Title: Please check if the “TEMPEST 1.0 Event Consortium” is suitable for the author list?

A consortium authorship is allowed by PLOSOne policy.

2. Abstract: The method and main results of the entire study are missing. The author needs to add more methods and applied results to understand the actual study analysis.

We have reworked the abstract significantly to bring up more details of the experimental methods and highlight the key findings from the study.

3. “Most studies on the conversion of coastal upland ecosystems to wetlands are observation-based, using a space for time substitution design that is ultimately limited in its ability to test hypotheses about the mechanisms that underlie ecosystem state change.” Need reference.

We have added two references to this sentence, one an example of a space-for-time substitution and one an article about key limitations of space-for-time substitutions.

4. “vegetation community structures” is this an appropriate word? Recheck this.

We have changed this to “community composition”.

5. The introduction section lacks an in-depth discussion of the current study, research method and literature review.

Based on this comment and several other comments from R1 and R3, we’ve reworked the introduction significantly with a focus on adding in more details of context in the literature (lines 91-108), the experimental design (lines 109-111) and motivation for the current study (lines 120-131).

6. Research novelty is also limited in understanding. Restructure the introduction section.

We have restructured the introduction with a focus on novelty of the presented work. Please see lines 101-108 and 120-131.

7. “We delivered 263 m3 of freshwater and 267 m3” try to improve your result sections.

We have worked to more cleanly separate methods and results, and rewritten the results section to more clearly emphasize key findings.

8. In-depth discussion of the result section is very limited.

We found this comment difficult to interpret as there is a lack of specificity for us to focus our revisions on. However, we reviewed the results section with this comment in mind, and added in more details in the biogeochemical section to align with results presented in Table 2. We have

also reorganized and rewritten large parts of the results section to increase clarity of variables discussed. We believe that to go further than this would lengthen the paper without adding additional meaningful insights to our data.

9. Limitations and future research directions need to be added after discussion.

We have added a future research direction & conclusions section on lines 553-577 and discuss limitations on lines 496-500.

10. The conclusion is missing in the study.

We have added a future research direction & conclusions section on lines 553-577.

The manuscript needs more attention and revision accordingly. Based on the current status of the manuscript, I must recommend extensive major revision before publication.

---

6. PLOS authors have the option to publish the peer review history of their article (what does this mean?). If published, this will include your full peer review and any attached files.

If you choose “no”, your identity will remain anonymous but your review may still be made public.

Do you want your identity to be public for this peer review? For information about this choice, including consent withdrawal, please see our Privacy Policy.

Reviewer #1: No

Reviewer #2: No

Reviewer #3: No

[NOTE: If reviewer comments were submitted as an attachment file, they will be attached to this email and accessible via the submission site. Please log into your account, locate the manuscript record, and check for the action link "View Attachments". If this link does not appear, there are no attachment files.]

While revising your submission, please upload your figure files to the Preflight Analysis and Conversion Engine (PACE) digital diagnostic tool, https://pacev2.apexcovantage.com/. PACE helps ensure that figures meet PLOS requirements. To use PACE, you must first register as a user. Registration is free. Then, login and navigate to the UPLOAD tab, where you will find detailed instructions on how to use the tool. If you encounter any issues or have any questions when using PACE, please email PLOS at figures@plos.org. Please note that Supporting Information files do not need this step.
