# Supplementary material for: Short-term coastal forest responses to a hurricane-scale freshwater and saltwater flooding experiment
Source: PLoS One. 2026 May 13;21(5):e0323584. doi: 10.1371/journal.pone.0323584 (PMC13170863; doi:10.1371/journal.pone.0323584)
Supplement: Attachment — Submitted filename: Response to Reviews 2 PONE.pdf [file pone.0323584.s008.pdf]

PONE-D-25-19393R1
Short-term coastal forest responses to a hurricane-scale freshwater and saltwater flooding experiment
PLOS One

Dear Dr. Myers-Pigg,

Thank you for submitting your manuscript to PLOS ONE. After careful consideration, we feel that it has merit but does not fully meet PLOS ONE's publication criteria as it currently stands. Therefore, we invite you to submit a revised version of the manuscript that addresses the points raised during the review process.

==============================
Dear authors,
Thank you for your efforts in addressing the reviewers comments previously.
Please refer to the comments of both reviewers, specially Reviewer 1, and address all the comments and suggestions.
==============================

Please submit your revised manuscript by Feb 27 2026 11:59PM. If you will need more time than this to complete your revisions, please reply to this message or contact the journal office at plosone@plos.org. When you're ready to submit your revision, log on to https://www.editorialmanager.com/pone/ and select the 'Submissions Needing Revision' folder to locate your manuscript file.

Please include the following items when submitting your revised manuscript:

- A letter that responds to each point raised by the academic editor and reviewer(s). You should upload this letter as a separate file labeled 'Response to Reviewers'.
- A marked-up copy of your manuscript that highlights changes made to the original version. You should upload this as a separate file labeled 'Revised Manuscript with Track Changes'.
- An unmarked version of your revised paper without tracked changes. You should upload this as a separate file labeled 'Manuscript'.

If you would like to make changes to your financial disclosure, please include your updated statement in your cover letter. Guidelines for resubmitting your figure files are available below the reviewer comments at the end of this letter.

If applicable, we recommend that you deposit your laboratory protocols in protocols.io to enhance the reproducibility of your results. Protocols.io assigns your protocol its own identifier (DOI) so that it can be cited independently in the future. For instructions see: https://journals.plos.org/plosone/s/submission-guidelines#loc-laboratory-protocols. Additionally, PLOS ONE offers an option for publishing peer-reviewed Lab Protocol

articles, which describe protocols hosted on protocols.io. Read more information on sharing protocols at https://plos.org/protocols?utm_medium=editorial-email&utm_source=authorletters&utm_campaign=protocols.

We look forward to receiving your revised manuscript.

Kind regards,

Miguel Inácio
Academic Editor
PLOS One

Journal Requirements:

If the reviewer comments include a recommendation to cite specific previously published works, please review and evaluate these publications to determine whether they are relevant and should be cited. There is no requirement to cite these works unless the editor has indicated otherwise.

[Note: HTML markup is below. Please do not edit.]

Reviewers' comments:

Reviewer's Responses to Questions

Comments to the Author

1. If the authors have adequately addressed your comments raised in a previous round of review and you feel that this manuscript is now acceptable for publication, you may indicate that here to bypass the “Comments to the Author” section, enter your conflict of interest statement in the “Confidential to Editor” section, and submit your "Accept" recommendation.

Reviewer #1: (No Response)

Reviewer #3: All comments have been addressed

---

2. Is the manuscript technically sound, and do the data support the conclusions?

The manuscript must describe a technically sound piece of scientific research with data that supports the conclusions. Experiments must have been conducted rigorously, with

appropriate controls, replication, and sample sizes. The conclusions must be drawn appropriately based on the data presented.

Reviewer #1: Partly

Reviewer #3: Yes

---

3. Has the statistical analysis been performed appropriately and rigorously?

Reviewer #1: Yes

Reviewer #3: Yes

---

4. Have the authors made all data underlying the findings in their manuscript fully available?

The PLOS Data policy requires authors to make all data underlying the findings described in their manuscript fully available without restriction, with rare exception (please refer to the Data Availability Statement in the manuscript PDF file). The data should be provided as part of the manuscript or its supporting information, or deposited to a public repository. For example, in addition to summary statistics, the data points behind means, medians and variance measures should be available. If there are restrictions on publicly sharing data—e.g. participant privacy or use of data from a third party—those must be specified.

Reviewer #1: Yes

Reviewer #3: Yes

---

5. Is the manuscript presented in an intelligible fashion and written in standard English?

PLOS ONE does not copyedit accepted manuscripts, so the language in submitted articles must be clear, correct, and unambiguous. Any typographical or grammatical errors should be corrected at revision, so please note any specific errors here.

Reviewer #1: Yes

Reviewer #3: Yes

---

6. Review Comments to the Author

Please use the space provided to explain your answers to the questions above. You may also include additional comments for the author, including concerns about dual publication, research ethics, or publication ethics. (Please upload your review as an attachment if it exceeds 20,000 characters)

Reviewer #1: (No Response)

Summary

Although authors have made significant changes that address some concerns in the initial manuscript, I am still not totally convinced that there were clear motivations or hypotheses for analysis of this first disturbance in isolation from the larger project. I might suggest that this study highlights its contribution to better understanding mechanisms underlying forest conversion; although this is mentioned, it is not clear throughout the different sections of the manuscript. Furthermore, although there were many (and very interesting) planned measurements for the larger TEMPEST project which are also reported here, it is not clear how they all connect to each other (mechanistically) or to the larger objectives of this manuscript. I have outlined general comments/suggestions for each section below as well as some more specific line by line comments.

Response: Thank you for your careful and thoughtful review of our manuscript. In response to these concerns, we focused on 1) clarifying the motivation for analyzing the first TEMPEST event in isolation, 2) tightening our hypotheses, and 3) strengthening the mechanistic framing throughout the manuscript. We also revised how individual measurements are presented and interpreted to explicitly connect them through a mechanistic cascade linking hydrology, soil biogeochemistry, root processes, and tree physiological responses. Detailed responses to each comment are provided below, including line numbers in the clean document where the edits can be found.

General comments

Introduction: More detailed mechanistic information about microbial processing/redox states/C quantity and quality/GHG production/tree physiology and how they connect is warranted. As is, it would be difficult for a non-specialist (without their own background knowledge) to understand how all of these measurements were relevant to the study questions. This was a concern I mentioned in the initial review that I don’t feel was addressed adequately. Additionally, I am still not clear on the motivation of the study. Management is mentioned, but not in enough detail (and only once) so it is not overly convincing as a motivator of this study.

Response: In response to this comment, we have increased the mechanistic descriptions in Table 1 and introduction. We now refer to Table 1 throughout the revised study motivation paragraph (Introduction, lines 96-115), where we describe the linkages between soil saturation and oxygen availability to microbial, root and tree physiological responses. We agree that management was not a clear motivator for this paper and have removed references to management in the current draft, focusing instead on understanding responses to novel exposure.

Methods: In addition to adding mechanistic detail to the introduction, the methods could be better contextualized/motivated by including statements that briefly explain why methods were used/how they connected to the broader goals of the study before diving into specifics.

Response: Thank you for this actionable suggestion. We have added several brief explanatory statements in the methods section about why each were chosen and how they connect to study goals. You will find these at the start of each subsection so the line numbers were not included here.

Example: *"Soil temperature, moisture content, and electrical conductivity (EC) are key drivers of vegetative and microbial stress (Table 1) [9]."*

Discussion: It seems to me that this section should be focused on dissecting if/how the results suggest provide insight into mechanisms of forest conversion (does a single disturbance set this process in motion by priming the response of a system to future disturbances?). This is addressed briefly in lines 679-683, but are not a current focus of the discussion. More mechanistic details connecting hydrology, soil biogeochemistry and tree physiology throughout would make for a more interesting discussion.

Response: We have reframed the discussion to explicitly follow the mechanistic cascade introduced in the Introduction (lines 96-100). The revised discussion now begins with observed changes in soil saturation and oxygen availability (new lines 443-479), then interprets how those changes influence belowground biogeochemistry (new lines 494-505), followed by downstream effects on root processes and tree physiological responses (new lines 506-520).

Line-by-line comments:

L79: Microbial activity is mentioned a number of times, but it isn't clear how it relates to your overarching goal of understanding changes in ecosystem function related to forest conversion? I would suggest stronger motivation for your interest in microbial community properties (you measure GHG fluxes but don't mention how they relate to microbial processing).

Response: We have increased mechanistic discussion of why the variables chosen are important throughout the introduction. Specifically, we now directly link this to soil ghg production on lines 82-84:

"Pulse flooding events can rapidly increase soil saturation and decrease oxygen availability on hourly timescales, driving shifts in the microbial metabolism underpinning soil greenhouse gas production."

L83-85: Management comes out of no where and doesn't seem to be a central part of the study. If management is going to be a central part of the motivation, it should be better explained (how does your study inform land management in the face of increased flooding/exposure to salinity)?

Response: We have removed this sentence and instead focus motivation on understanding responses to novel flooding and salinity exposure.

L95: I would think that soil carbon stocks are large and therefore slow to change in response to disturbance (I would expect soil C stocks to change after aboveground mortality).

Response: This sentence has been reworked in response to previous comments, and this statement no longer remains in the revised manuscript.

L100: Im not sure what you mean by "solubility or reactivity of key nutrients" and also not sure how this relates to GHG production. I would think the shifts in GHG production are driven by changes in redox conditions and C availability rather than in nutrient pools.

Response: Thanks for pointing out this ambiguous statement. This sentence now reads:

L72-74: ***"Changes may be immediate, driven by shifts in the availability of oxygen and key substrates [13,14], or lagged, as plant or soil microbial communities become more stressed over time [9]."***

L122-127: I agree that the initial properties of a system will affect its response to disturbance but Im not sure this motivates the present investigation of ecosystem response to a single disturbance (especially since this study only investigates one system and doesn't compare many different systems with different ecosystem properties).

Response: We appreciate the reviewer's point and agree that differences among systems are critical for understanding broader patterns of coastal ecosystem response. Our objective is not to compare transitions across sites, but to determine whether a first, novel flooding event is sufficient to generate short-term hydrologic

and biogeochemical conditions that potentially influence how this system could respond to subsequent disturbances. We have clarified this motivation in the Introduction (lines 94-96) and in the Discussion (starting on line 443).

L131-132: If this study only includes data from after an initial disturbance, the hypothesis statement shouldn't include anything about successive exposures – this isn't testable.

Response: We agree with this statement and have further tightened our hypotheses. The revised hypotheses now focus exclusively on responses within the 48-hour window following the first flooding event:

L104-113: "We hypothesize that hydrologic state variables (e.g., soil moisture and groundwater levels) will respond to the event immediately but transiently, followed by muted biogeochemical (e.g., soil greenhouse gases, soil porewaters) responses, and finally minimal vegetation (e.g., tree sap flux density) responses in the 48 hours following the novel flooding event. Further, we hypothesize that the response of the upland forest ecosystem to freshwater flooding and saline flooding will be similar for hydrologic variables, while salinity changes from estuarine water flooding will have a greater impact on biogeochemical and vegetation variables in the 48 hours following flooding –as compared to freshwater flooding – that would have negligible impacts on biogeochemical and vegetation variables."

L164-166: But why? This is a good opportunity to connect to the bigger picture – why do we care about the response of a system to a single exposure when we are ultimately interested in forest conversion which occurs after repeated exposure?

Response: We have modified this sentence in response to this comment; it now reads:

L113-115: "By isolating ecosystem responses during and immediately following the first, novel flooding event, we present short-term shifts that may occur, establishing antecedent conditions that could shape future responses."

L572-574: I believe this is a contribution of the overarching TEMPEST project, but analysis of the initial disturbance in this manuscript does not help us to characterize "thresholds of ecosystem state change from repeated flooding and salinity exposure"

Response: We agree that this paragraph read as motivation for the overarching project and not for this specific study. We have reworked this paragraph and this sentence has been removed.

L624-626: Im not sure this topic sentence makes sense given that the rest of the paragraph talks about changes in properties in response to flooding.

Response: Thanks for pointing this out. We have integrated this paragraph into the preceding one and removed this sentence.

L642-654: I thought it was interesting that microbial CH4 fluxes increased after saltwater flooding but decreased (then increased) after freshwater flooding. There is no discussion about this or potential implications for GHG emissions from these systems.

Response: We note in the current discussion that physical factors could be clouding any interpretation of the short term GHG fluxes after flooding. While we agree these are interesting trends, high spatial variability (denoted by the vertical lines in the plots) make it difficult to give attribution to the trends observed. Therefore, in response to this comment we've changed the topic sentence of this paragraph to make this point clearer but leave the rest of this part of the discussion as is. Topic sentence now reads:

L494-497: "The long-term implications of the transient changes in CO_2 and CH_4 fluxes observed are harder to discern because they are determined by interacting physical and biological factors and display high spatial variability throughout the study period."

L652-654: This statement is confusing. The whole manuscript is based around investigation of a short term response; it is not clear why long term responses are brought up here specifically. Is there anything interesting that can be gathered from the data (rather than just suggesting short and long term dynamics are different?)

Response: This sentence meant to correlate short term responses with physical factors and longer term with biological responses. We recognize that the statement was ambiguous as written and have adjusted the sentence to more clearly make that comparison. It now reads:

L503-505: "Further field observations coupled with lab experiments are needed to isolate the mechanisms attributable to short-term physical factors versus biological responses to freshwater and saltwater flooding."

L659-661: There is no context provided for readers to understand these concepts (sap flow, root influence on CH4 fluxes)

Response: In response to previous comments, we have included increased rationale for these concepts in the intro (lines 98-100) and the methods sections (sap flow: lines 243-245; root CH4: 229-231). We also increase context for why the focus on these variables in the beginning of the paragraph:

L508-513: "Although vegetation structural responses would be outside of our 48-hour response window, tree sap flow velocity and root greenhouse gas fluxes are governed by immediate physical and physiological constraints on water and oxygen availability that may respond within our 48-hour response window [9,12]. Therefore, we examined the responses of tree sap flow velocity, root respiration, and root-influenced soil CH_4 flux to our experimental

flooding event, with the understanding that the influence of roots on CH_4 flux would operate indirectly through physical or microbial processes."

L736-741: I think the discussion should focus on if the data from this first disturbance event improve our mechanistic understanding of forest conversion, rather than just setting up papers to come from the larger TEMPEST project.

Response: We have edited this part of the discussion heavily with a focus on synthesizing and reiterating the importance of the current study. Please see the revised section lines 534-553.

Reviewer #3: The authors have revised their work properly in response to the previous comments, but some minor modifications are still needed for this manuscript.
1. Why are references added in the conclusion section? The conclusion must be from your own study and analysis results.
2. If those references are for future recommendations, please separate the future recommendations and conclusion.
Best of Luck

Response: We thank the reviewer for this suggestion and agree that conclusions should primarily synthesize insights from the present study rather than introduce new evidence. According to PLOS ONE's manuscript guidelines, the conclusions section should summarize principal findings and their relevance in a concise manner without introducing new arguments or evidence that were not previously discussed in the results or discussion. We have revised this section to remove citations that were not directly tied to interpretation of our results; any remaining references are included only to contextualize our findings. We also have reworked the text in this section in response to another reviewer's comment, further reducing the forward-looking portion of the section.

---

7. PLOS authors have the option to publish the peer review history of their article (what does this mean?). If published, this will include your full peer review and any attached files.

If you choose "no", your identity will remain anonymous but your review may still be made public.

Do you want your identity to be public for this peer review? For information about this choice, including consent withdrawal, please see our Privacy Policy.

Reviewer #1: No

Reviewer #3: No

[NOTE: If reviewer comments were submitted as an attachment file, they will be attached to this email and accessible via the submission site. Please log into your account, locate the manuscript record, and check for the action link "View Attachments". If this link does not appear, there are no attachment files.]

To ensure your figures meet our technical requirements, please review our figure guidelines: https://journals.plos.org/plosone/s/figures

You may also use PLOS's free figure tool, NAAS, to help you prepare publication quality figures: https://journals.plos.org/plosone/s/figures#loc-tools-for-figure-preparation.

NAAS will assess whether your figures meet our technical requirements by comparing each figure against our figure specifications.

In compliance with data protection regulations, you may request that we remove your personal registration details at any time. (Remove my information/details). Please contact the publication office if you have any questions.
