# Supplementary material for: Short-term coastal forest responses to a hurricane-scale freshwater and saltwater flooding experiment
Source: PLoS One. 2026 May 13;21(5):e0323584. doi: 10.1371/journal.pone.0323584 (PMC13170863; doi:10.1371/journal.pone.0323584)
Supplement: Attachment — Submitted filename: 251225_PLOS ONE_resumbission_review.docx [file pone.0323584.s007.docx]

**Summary**

Although authors have made significant changes that address some concerns in the initial manuscript, I am still not totally convinced that there were clear motivations or hypotheses for analysis of this first disturbance in isolation from the larger project. I might suggest that this study highlights its contribution to better understanding mechanisms underlying forest conversion; although this is mentioned, it is not clear throughout the different sections of the manuscript. Furthermore, although there were many (and very interesting) planned measurements for the larger TEMPEST project which are also reported here, it is not clear how they all connect to each other (mechanistically) or to the larger objectives of this manuscript. I have outlined general comments/suggestions for each section below as well as some more specific line by line comments.

**General comments**

Introduction: More detailed mechanistic information about microbial processing/redox states/C quantity and quality/GHG production/tree physiology and how they connect is warranted. As is, it would be difficult for a non-specialist (without their own background knowledge) to understand how all of these measurements were relevant to the study questions. This was a concern I mentioned in the initial review that I don’t feel was addressed adequately. Additionally, I am still not clear on the motivation of the study. Management is mentioned, but not in enough detail (and only once) so it is not overly convincing as a motivator of this study.

Methods: In addition to adding mechanistic detail to the introduction, the methods could be better contextualized/motivated by including statements that briefly explain why methods were used/how they connected to the broader goals of the study before diving into specifics.

Discussion: It seems to me that this section should be focused on dissecting if/how the results suggest provide insight into mechanisms of forest conversion (does a single disturbance set this process in motion by priming the response of a system to future disturbances?). This is addressed briefly in lines 679-683, but are not a current focus of the discussion. More mechanistic details connecting hydrology, soil biogeochemistry and tree physiology throughout would make for a more interesting discussion.

**Line-by-line comments:**

L79: Microbial activity is mentioned a number of times, but it isn’t clear how it relates to your overarching goal of understanding changes in ecosystem function related to forest conversion? I would suggest stronger motivation for your interest in microbial community properties (you measure GHG fluxes but don’t mention how they relate to microbial processing).

L83-85: Management comes out of no where and doesn’t seem to be a central part of the study. If management is going to be a central part of the motivation, it should be better explained (how does your study inform land management in the face of increased flooding/exposure to salinity)?

L95: I would think that soil carbon stocks are large and therefore slow to change in response to disturbance (I would expect soil C stocks to change after aboveground mortality).

L100: Im not sure what you mean by “solubility or reactivity of key nutrients” and also not sure how this relates to GHG production. I would think the shifts in GHG production are driven by changes in redox conditions and C availability rather than in nutrient pools.

L122-127: I agree that the initial properties of a system will affect its response to disturbance but Im not sure this motivates the present investigation of ecosystem response to a single disturbance (especially since this study only investigates one system and doesn’t compare many different systems with different ecosystem properties).

L131-132: If this study only includes data from after an initial disturbance, the hypothesis statement shouldn’t include anything about successive exposures – this isn’t testable.

L164-166: But why? This is a good opportunity to connect to the bigger picture – why do we care about the response of a system to a single exposure when we are ultimately interested in forest conversion which occurs after repeated exposure?

L572-574: I believe this is a contribution of the overarching TEMPEST project, but analysis of the initial disturbance in this manuscript does not help us to characterize “thresholds of ecosystem state change from repeated flooding and salinity exposure”

L624-626: Im not sure this topic sentence makes sense given that the rest of the paragraph talks about changes in properties in response to flooding.

L642-654: I thought it was interesting that microbial CH4 fluxes increased after saltwater flooding but decreased (then increased) after freshwater flooding. There is no discussion about this or potential implications for GHG emissions from these systems.

L652-654: This statement is confusing. The whole manuscript is based around investigation of a short term response; it is not clear why long term responses are brought up here specifically. Is there anything interesting that can be gathered from the data (rather than just suggesting short and long term dynamics are different?)

L659-661: There is no context provided for readers to understand these concepts (sap flow, root influence on CH4 fluxes)

L736-741: I think the discussion should focus on if the data from this first disturbance event improve our mechanistic understanding of forest conversion, rather than just setting up papers to come from the larger TEMPEST project.
