# Supplementary material for: Short-term coastal forest responses to a hurricane-scale freshwater and saltwater flooding experiment
Source: PLoS One. 2026 May 13;21(5):e0323584. doi: 10.1371/journal.pone.0323584 (PMC13170863; doi:10.1371/journal.pone.0323584)
Supplement: Attachment — Submitted filename: PONE Reviewer Comments 3rd revisions.docx [file pone.0323584.s009.docx]

PONE-D-25-19393R2

Short-term coastal forest responses to a hurricane-scale freshwater and saltwater flooding experiment

PLOS One

Dear Dr. Myers-Pigg,

Thank you for submitting your manuscript to PLOS ONE. After careful consideration, we feel that it has merit but does not fully meet PLOS ONE’s publication criteria as it currently stands. Therefore, we invite you to submit a revised version of the manuscript that addresses the points raised during the review process.

==============================
**ACADEMIC EDITOR:**

**Dear authors, thank you for the thorough review in response to the previous reviewers' comments. 
Please refer to Reviewer 1's comments.**

**We have addressed all Reviewer comments and respond in bold blue text below. Thank you for your consideration of our revised manuscript.**

==============================

Please submit your revised manuscript by May 15 2026 11:59PM. If you will need more time than this to complete your revisions, please reply to this message or contact the journal office at [plosone@plos.org](mailto:plosone@plos.org). When you're ready to submit your revision, log on to <https://www.editorialmanager.com/pone/> and select the 'Submissions Needing Revision' folder to locate your manuscript file.

Please include the following items when submitting your revised manuscript:

- A letter that responds to each point raised by the academic editor and reviewer(s). You should upload this letter as a separate file labeled 'Response to Reviewers'.
- A marked-up copy of your manuscript that highlights changes made to the original version. You should upload this as a separate file labeled 'Revised Manuscript with Track Changes'.
- An unmarked version of your revised paper without tracked changes. You should upload this as a separate file labeled 'Manuscript'.

If you would like to make changes to your financial disclosure, please include your updated statement in your cover letter. Guidelines for resubmitting your figure files are available below the reviewer comments at the end of this letter.

If applicable, we recommend that you deposit your laboratory protocols in protocols.io to enhance the reproducibility of your results. Protocols.io assigns your protocol its own identifier (DOI) so that it can be cited independently in the future. For instructions see: [https://journals.plos.org/plosone/s/submission-guidelines#loc-laboratory-protocols](https://gcc02.safelinks.protection.outlook.com/?url=https%3A%2F%2Ftrack.editorialmanager.com%2FCL0%2Fhttps%3A%252F%252Fjournals.plos.org%252Fplosone%252Fs%252Fsubmission-guidelines%2523loc-laboratory-protocols%2F1%2F010f019d46e82e2d-98ae45de-b601-47ab-a6cb-afc3211c3dd9-000000%2FU-WRvf2MatqSCmKkux6rJa6A6ON_BRjhbRtZRd9wP40%3D257&data=05%7C02%7Callison.myers-pigg%40pnnl.gov%7C8bbc7f3c31424cc1ea4208de8f97c5a8%7Cd6faa5f90ae240338c0130048a38deeb%7C0%7C0%7C639106079174408571%7CUnknown%7CTWFpbGZsb3d8eyJFbXB0eU1hcGkiOnRydWUsIlYiOiIwLjAuMDAwMCIsIlAiOiJXaW4zMiIsIkFOIjoiTWFpbCIsIldUIjoyfQ%3D%3D%7C0%7C%7C%7C&sdata=KjfYllH5%2FywO1LZiJFbeweN8GBFHyB3RVtiPGqH02s4%3D&reserved=0). Additionally, PLOS ONE offers an option for publishing peer-reviewed Lab Protocol articles, which describe protocols hosted on protocols.io. Read more information on sharing protocols at [https://plos.org/protocols?utm_medium=editorial-email&utm_source=authorletters&utm_campaign=protocols](https://gcc02.safelinks.protection.outlook.com/?url=https%3A%2F%2Ftrack.editorialmanager.com%2FCL0%2Fhttps%3A%252F%252Fplos.org%252Fprotocols%253Futm_medium%3Deditorial-email%2526utm_source%3Dauthorletters%2526utm_campaign%3Dprotocols%2F1%2F010f019d46e82e2d-98ae45de-b601-47ab-a6cb-afc3211c3dd9-000000%2FzSDX9FUAMPsf62lKmWcQ3MklxEqh8P5lB3566Ay4Q5w%3D257&data=05%7C02%7Callison.myers-pigg%40pnnl.gov%7C8bbc7f3c31424cc1ea4208de8f97c5a8%7Cd6faa5f90ae240338c0130048a38deeb%7C0%7C0%7C639106079174436565%7CUnknown%7CTWFpbGZsb3d8eyJFbXB0eU1hcGkiOnRydWUsIlYiOiIwLjAuMDAwMCIsIlAiOiJXaW4zMiIsIkFOIjoiTWFpbCIsIldUIjoyfQ%3D%3D%7C0%7C%7C%7C&sdata=LYQMKdYNeu2is0XXzQ5Lmzrx0ixrcxvhk%2F9A%2FlWCDzw%3D&reserved=0).

As the corresponding author, your ORCID iD is verified in the submission system and will appear in the published article. PLOS supports the use of ORCID, and we encourage all coauthors to register for an ORCID iD and use it as well. Please encourage your coauthors to verify their ORCID iD within the submission system before final acceptance, as unverified ORCID iDs will not appear in the published article. *Only* the individual author can complete the verification step; PLOS staff *cannot* verify ORCID iDs on behalf of authors.

We look forward to receiving your revised manuscript.

Kind regards,

Miguel Inácio
Academic Editor
PLOS One

Journal Requirements:

1. If the reviewer comments include a recommendation to cite specific previously published works, please review and evaluate these publications to determine whether they are relevant and should be cited. There is no requirement to cite these works unless the editor has indicated otherwise. 

2. Please review your reference list to ensure that it is complete and correct. If you have cited papers that have been retracted, please include the rationale for doing so in the manuscript text, or remove these references and replace them with relevant current references. Any changes to the reference list should be mentioned in the rebuttal letter that accompanies your revised manuscript. If you need to cite a retracted article, indicate the article’s retracted status in the References list and also include a citation and full reference for the retraction notice.

[Note: HTML markup is below. Please do not edit.]

Reviewers' comments:

Reviewer's Responses to Questions

**Comments to the Author**

1. If the authors have adequately addressed your comments raised in a previous round of review and you feel that this manuscript is now acceptable for publication, you may indicate that here to bypass the “Comments to the Author” section, enter your conflict of interest statement in the “Confidential to Editor” section, and submit your "Accept" recommendation.

Reviewer #1: (No Response)

Reviewer #3: All comments have been addressed

2. Is the manuscript technically sound, and do the data support the conclusions?

The manuscript must describe a technically sound piece of scientific research with data that supports the conclusions. Experiments must have been conducted rigorously, with appropriate controls, replication, and sample sizes. The conclusions must be drawn appropriately based on the data presented.

Reviewer #1: Yes

Reviewer #3: Yes

3. Has the statistical analysis been performed appropriately and rigorously?

Reviewer #1: Yes

Reviewer #3: Yes

4. Have the authors made all data underlying the findings in their manuscript fully available?

The [PLOS Data policy](https://gcc02.safelinks.protection.outlook.com/?url=http%3A%2F%2Ftrack.editorialmanager.com%2FCL0%2Fhttp%3A%252F%252Fwww.plosone.org%252Fstatic%252Fpolicies.action%2523sharing%2F1%2F010f019d46e82e2d-98ae45de-b601-47ab-a6cb-afc3211c3dd9-000000%2FcCyQ6g2BQiRxOwQghmf4nUyDtdrmpFEjnyFRV053P40%3D257&data=05%7C02%7Callison.myers-pigg%40pnnl.gov%7C8bbc7f3c31424cc1ea4208de8f97c5a8%7Cd6faa5f90ae240338c0130048a38deeb%7C0%7C0%7C639106079174453432%7CUnknown%7CTWFpbGZsb3d8eyJFbXB0eU1hcGkiOnRydWUsIlYiOiIwLjAuMDAwMCIsIlAiOiJXaW4zMiIsIkFOIjoiTWFpbCIsIldUIjoyfQ%3D%3D%7C0%7C%7C%7C&sdata=dHgQdw8nZAzJfMWohrr7JF1nGn0NaT1NQuBEgRJe4DI%3D&reserved=0) requires authors to make all data underlying the findings described in their manuscript fully available without restriction, with rare exception (please refer to the Data Availability Statement in the manuscript PDF file). The data should be provided as part of the manuscript or its supporting information, or deposited to a public repository. For example, in addition to summary statistics, the data points behind means, medians and variance measures should be available. If there are restrictions on publicly sharing data—e.g. participant privacy or use of data from a third party—those must be specified.

Reviewer #1: Yes

Reviewer #3: Yes

5. Is the manuscript presented in an intelligible fashion and written in standard English?

PLOS ONE does not copyedit accepted manuscripts, so the language in submitted articles must be clear, correct, and unambiguous. Any typographical or grammatical errors should be corrected at revision, so please note any specific errors here.

Reviewer #1: Yes

Reviewer #3: Yes

6. Review Comments to the Author

Please use the space provided to explain your answers to the questions above. You may also include additional comments for the author, including concerns about dual publication, research ethics, or publication ethics. (Please upload your review as an attachment if it exceeds 20,000 characters)

Reviewer #1: Summary
Authors edits in were extremely thorough and I found this version of the manuscript to be significantly improved. Some final thoughts summarized below:
For the introduction, I think there is some room to clarify specific sentences (called out in line by line comments), but generally, the study is effectively introduced!

**Thank you for your careful assessment of this manuscript. We have addressed all comments below.**

For the discussion, I appreciated how the topic sentences of each paragraph made the flow of the story really clear! I called out some specific spots in the discussion where I think that 1) results could reiterated or 2) interpretation/synthesis could be stronger.

**Thank you for this suggestion and we have addressed your additional suggestions in the line by line comments below.**

Line by line comments:
Lines 67-70: Long and some confusing structure – could be revised to increase clarity

**We have split the sentence into two based on the reviewer comment to improve clarity. This now reads:**

**“While thresholds of flooding and salinity exposure associated with coastal forest mortality have been observed [6–8], the underlying mechanisms remain unclear. Hypothesized mechanisms involve a complex mixture of above and belowground processes [9], which may even be triggered by a single, novel salinity exposure [10].”**

L72: I think this statement could be strengthened by clarifying changes in what?

**This sentence refers to the changes in the previous sentence. We’ve modified this to clarify as follows:**

**“The effects of flooding and salinity exposure on biogeochemical and ecological processes may be immediate, driven by shifts in the availability of oxygen and key substrates [13,14], or lagged, as plant or soil microbial communities become more stressed over time [9].”**

L 87: Clarify what you mean by “initial soil properties”. Are you talking about prior exposure to flooding/salinity?

**Yes, this is alluding to antecedent conditions but also overall soil properties, such as clay content, etc. We’ve clarified by modifying this to read “…antecedent conditions and soil properties”**

L96-100: I like the idea of this sentence, but it reads awkwardly and ends up being difficult to follow. Maybe rephrase?

**We split the sentence and revised it for clarity. It now reads:**

**“We focus on a suite of variables that capture the mechanistic cascade by which flooding and salinity alter tree and soil functions [9]. In this framework, soil saturation alters oxygen availability, potentially reshaping belowground microbial processes, root processes, and tree physiological functions (Table 1).”**

L108-113: I think this hypothesis statement could be condensed to make your meaning more clear.

**We have revised and condensed our hypotheses to increase clarity for the reader:**

**“We hypothesize that hydrologic state variables (e.g., soil moisture, groundwater levels) will respond immediately but transiently to flooding, followed by more muted biogeochemical responses (e.g., soil greenhouse gases, soil porewaters), and minimal vegetation responses (e.g., tree sap flow velocity) within 48 hours. We further hypothesize that hydrologic responses will be similar between freshwater and saline flooding, but that salinity exposure will initiate stronger biogeochemical and vegetative responses than freshwater flooding over the same period.”**

L454-455: Might be worth a brief explanation of ‘electromigration’ because this is the only time it is mentioned in the paper. As a non-expert, Im not sure if this is an important takehome or not!

**We have modified the sentence to describe the phenomenon of “electromigration”:**

**“We attribute the observed increase in soil electrical conductivity in the freshwater plot to enhanced ion mobility (e.g. increased electromigration) associated with higher soil water content rather than an increase in salinity.”**

L468-471: Awkward structure – consider editing. Additionally, Im wondering if there is any interpretation of this finding (saltwater inundation decreases concentrations of high molecular weight compounds in DOC, but freshwater inundation does the opposite)?

**We have edited the structure of this sentence and added in a sentence on the interpretation of the finding, see below:**

**“The slight increase in spectral slope ratio in the saltwater plot suggests preferential removal of higher molecular weight compounds from the DOC pool during saline flooding, while freshwater flooding may retain these compounds [47]. This change in DOC quality may influence the availability of the porewater DOC to be utilized by microbial communities, as a higher slope ratio is usually associated with more degraded, lower-molecular weight organic matter [47].”**

L473: Not sure what you mean by “quantitatively important anaerobic biogeochemical cycles” – the meaning of this interpretation could be clarified.

**We’ve modified this sentence to the following to increase clarity and make the interpretation easier to follow:**

**The relative decline in soil O_2_ was surprising for a short-term event and suggests that this upland forest, with typically aerobic soils, may shift quite rapidly toward anaerobic biogeochemical processes as the duration of events lengthens from several hours to several days [14].**

L480-505: Final sentences in these two paragraphs negates any of your findings – kind of deflates any takeaway points. I wonder if the structure could be shifted so that you acknowledge shortcomings, and then highlight important takeaways despite these shortcomings?

**We believe that the reviewer is referring to the following two sentences:**

1. **“ Therefore, coupling the large-scale field manipulation with model simulations [49] and laboratory experiments [11] will be key to further disentangling mechanistic relationships and generate predictive understanding [50].”**
2. **“Further field observations coupled with lab experiments are needed to isolate the mechanisms attributable to short-term physical factors versus biological responses to freshwater and saltwater flooding.”**

**Based on this, we’ve revised the structure of this section slightly, removed the two sentences in question, and added a short qualifying section in the conclusions section on lines 555-557:**

**“ Future research coupling large-scale field manipulation with model simulations [49] and laboratory experiments [11] will be key to further disentangling mechanistic relationships and generate predictive understanding [50].”**

498-499: I would suggest spelling out mechanism more explicitly – say the relationship between soil moisture content and CH4 diffusion rather than just mentioning that there is a relationship.

**This sentence has been revised to explicitly talk about the mechanism:**

**“CH_4_ uptake by soils is strongly influenced by soil moisture content, as higher water content limits CH_4_ diffusion from the atmosphere into the soil, and constrains microbial utilization [53–55]; the higher water content in the freshwater plot could therefore explain the lower CH_4_ response compared to the saltwater plot.”**

L500: I wouldn’t assume that readers remember differences in CH4 responses between plots. This is actually a general comment about the discussion. In some cases, think I could be helpful to add reminders about general findings before diving into interpretation.

**We have revised this sentence completely based on the previous comment. Please refer to the response above for the changes made. Additionally, we have read the entire discussion section with the second part of the comment in mind and made additional clarifying statements reiterating on our findings when applicable.**

L524-520: Is there any take-home message from these results? How should readers be thinking about the potential for pulses of fresh/saltwater to affect veg?

**We have added the following sentence at the end of the paragraph to address this comment”**

**“Together, these results confirm that short-term hydrologic events can induce measurable changes in plant physiological functions, with freshwater additions temporarily removing water limitation and salinity exposure potentially inducing stress responses.”**

L523-532: Im not overly convinced that there was enough information gathered from the rain event to support your ideas here. Maybe ideas just need to be clarified further?

**We have revised this section based on the reviewer’s comment. It now reads:**

**“Transient changes in key drivers of tree mortality, such as soil moisture and oxygen levels, can influence long-term responses by changing baseline conditions, affecting the ability of the system to respond to future perturbations [24]. Evidence for such legacy effects is suggested by the response of the system to a rain event that occurred < 24 hours after the TEMPEST treatments ended. Soil O_2_ concentrations in the saltwater plot had only partially recovered (to about half of atmospheric saturation) before rain began, while concentrations in the control plot started at full saturation. Consequently, soil O_2_ concentrations were depleted much faster in the saltwater than control plots post-event, consistent with the differences in initial conditions due to the flooding event. While this single event cannot confirm causality, it suggests that even the brief anaerobic conditions due to the flooding event could alter antecedent conditions in ways that influence responses to subsequent disturbances. Repeated episodic events may amplify these effects, potentially driving changes in belowground redox, carbon, and nutrient cycling that may become more pronounced with repeated episodic events [60,61] and inducing shifts in key processes regulating vegetative dynamics leading to plant stress and mortality [9].”**

Reviewer #3: The authors are revised the manuscript based on the comments. Therefore, I must recommend accept manuscript for publication

**Thank you!**

7. PLOS authors have the option to publish the peer review history of their article ([what does this mean?](https://gcc02.safelinks.protection.outlook.com/?url=https%3A%2F%2Ftrack.editorialmanager.com%2FCL0%2Fhttps%3A%252F%252Fjournals.plos.org%252Fplosone%252Fs%252Feditorial-and-peer-review-process%2523loc-peer-review-history%2F1%2F010f019d46e82e2d-98ae45de-b601-47ab-a6cb-afc3211c3dd9-000000%2FXjzfI9cO8vgD6tmxWfPZX3fYXZPbQmDCsb4arnfk_Q4%3D257&data=05%7C02%7Callison.myers-pigg%40pnnl.gov%7C8bbc7f3c31424cc1ea4208de8f97c5a8%7Cd6faa5f90ae240338c0130048a38deeb%7C0%7C0%7C639106079174466795%7CUnknown%7CTWFpbGZsb3d8eyJFbXB0eU1hcGkiOnRydWUsIlYiOiIwLjAuMDAwMCIsIlAiOiJXaW4zMiIsIkFOIjoiTWFpbCIsIldUIjoyfQ%3D%3D%7C0%7C%7C%7C&sdata=MacZgap7f2cBfWgVMWPCDkneZ0XNf4B%2Fjygr3LKTcVc%3D&reserved=0)). If published, this will include your full peer review and any attached files.


If you choose “no”, your identity will remain anonymous but your review may still be made public.


**Do you want your identity to be public for this peer review?** For information about this choice, including consent withdrawal, please see our [Privacy Policy](https://gcc02.safelinks.protection.outlook.com/?url=https%3A%2F%2Ftrack.editorialmanager.com%2FCL0%2Fhttps%3A%252F%252Fwww.plos.org%252Fprivacy-policy%2F1%2F010f019d46e82e2d-98ae45de-b601-47ab-a6cb-afc3211c3dd9-000000%2FkzAJmF2Wa1MyayLfY9JHZEDLkMssk3j6Qv-aMmAjA20%3D257&data=05%7C02%7Callison.myers-pigg%40pnnl.gov%7C8bbc7f3c31424cc1ea4208de8f97c5a8%7Cd6faa5f90ae240338c0130048a38deeb%7C0%7C0%7C639106079174478971%7CUnknown%7CTWFpbGZsb3d8eyJFbXB0eU1hcGkiOnRydWUsIlYiOiIwLjAuMDAwMCIsIlAiOiJXaW4zMiIsIkFOIjoiTWFpbCIsIldUIjoyfQ%3D%3D%7C0%7C%7C%7C&sdata=lkvRuHxUAn9knKDugrQPaCjxwJethVpV5S6JuJJUYAU%3D&reserved=0).

Reviewer #1: No

Reviewer #3: No
